# 482. Long-Term Outcomes of Coronavirus disease 2019 and Risk Factors for Prolonged SARS-CoV-2 Infection in Lymphoma Patients: Multicenter, Retrospective Cohort Study

**DOI:** 10.1093/ofid/ofad500.552

**Published:** 2023-11-27

**Authors:** Jung Ah Lee, Min Han, Sangmin Ahn, Yongseop Lee, Joon-sup Yeom, Jun Yong Choi, Nam Su Ku, Su Jin Jeong, Jung Ho Kim, Jin Seok Kim, Haerim Chung, Hyunsoo Cho, Yu Ri Kim, Jin Young Ahn

**Affiliations:** Yonsei University College of Medicine, Seoul, Seoul-t'ukpyolsi, Republic of Korea; Yonsei University School of Medicine, Seoul, Seoul-t'ukpyolsi, Republic of Korea; Yonsei University College of Medicine, Seoul, Seoul-t'ukpyolsi, Republic of Korea; Yonsei University School of Medicine, Seoul, Seoul-t'ukpyolsi, Republic of Korea; Division of Infectious Diseases, Department of Internal Medicine, Yonsei University College of Medicine, Seoul, Seoul-t'ukpyolsi, Republic of Korea; Yonsei University College of Medicine, Seoul, Seoul-t'ukpyolsi, Republic of Korea; Division of Infectious Diseases, Department of Internal Medicine, Yonsei University College of Medicine, Seoul, Seoul-t'ukpyolsi, Republic of Korea; Yonsei University College of Medicine, Seoul, Seoul-t'ukpyolsi, Republic of Korea; Yonsei University College of Medicine, Seoul, Seoul-t'ukpyolsi, Republic of Korea; Yonsei University College of Medicine, Seoul, Seoul-t'ukpyolsi, Republic of Korea; Yonsei University College of Medicine, Seoul, Seoul-t'ukpyolsi, Republic of Korea; Yonsei University College of Medicine, Seoul, Seoul-t'ukpyolsi, Republic of Korea; Yonsei University College of Medicine, Seoul, Seoul-t'ukpyolsi, Republic of Korea; Yonsei University College of Medicine, Seoul, Seoul-t'ukpyolsi, Republic of Korea

## Abstract

**Background:**

In lymphoma patients, SARS-CoV-2 is often detected continuously even after a sufficient amount of time has passed since being confirmed with COVID-19. We aim to describe the long-term outcomes of COVID-19 and identify the risk factors for prolonged SARS-CoV-2 infection in lymphoma patients.

**Methods:**

A multicenter retrospective cohort study was conducted on patients receiving treatment for lymphoma at three tertiary hospitals in South Korea who were quarantined after being diagnosed with COVID-19 by polymerase chain reaction (PCR) testing or antigen test from August 2021 to July 2022. Prolonged COVID-19 is defined as a case where PCR remains positive even after 30 days of COVID-19 confirmation or when COVID-19 pneumonia occurs or worsens after 30 days of COVID-19 confirmation.

**Results:**

A total of 77 lymphoma patients with COVID-19 were studied. Among them, 24 (31%) patients were classified as prolonged COVID-19 and seven patients (9.1%) died within 90 days after the diagnosis of COVID-19. (Table 1) Prolonged COVID-19 was more frequently identified in follicular lymphoma patients with a history of bendamustine and rituximab (BR) treatment (22.6% vs. 50.0%, p=0.016), especially those who maintained rituximab maintenance treatment after BR treatment (11.3% vs. 37.5%, p=0.012). (Table 2) At the time of initial COVID-19 diagnosis, the 'prolonged COVID-19 group' did not show clinically significant differences compared to the non-prolonged COVID group. (Table 3) Multivariable analysis showed that BR treatment followed by rituximab maintenance is one of the risk factors for persistent PCR positivity (OR 3.375, 95% CI 1.104-10.317, p=0.033), delayed or persistent pneumonia (OR 8.833, 95% CI 2.527-30.879, p=0.001), and COVID-19 related admission after quarantine period (OR 11.1, 95% CI 2.758-44.678, p=0.001). Prolonged COVID-19 had an impact on the delay of cancer treatment but did not increase the risk of 90-day or 180-day mortality significantly. (Table 4)

**Figure:**
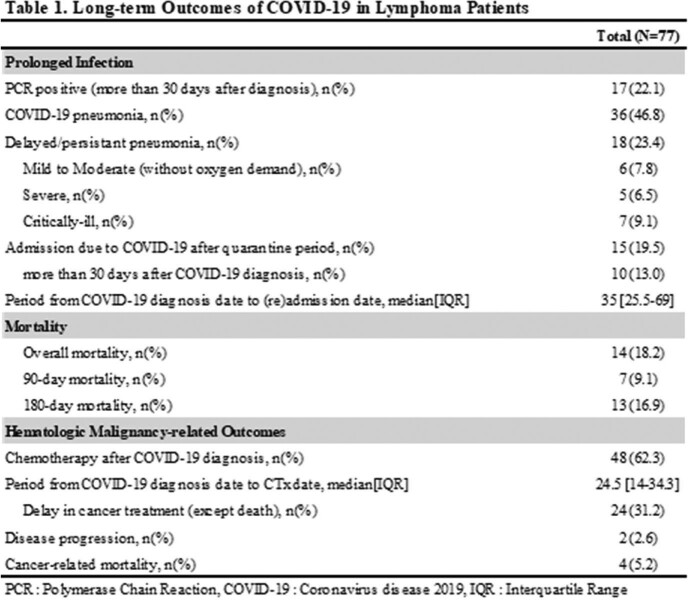

**Figure:**
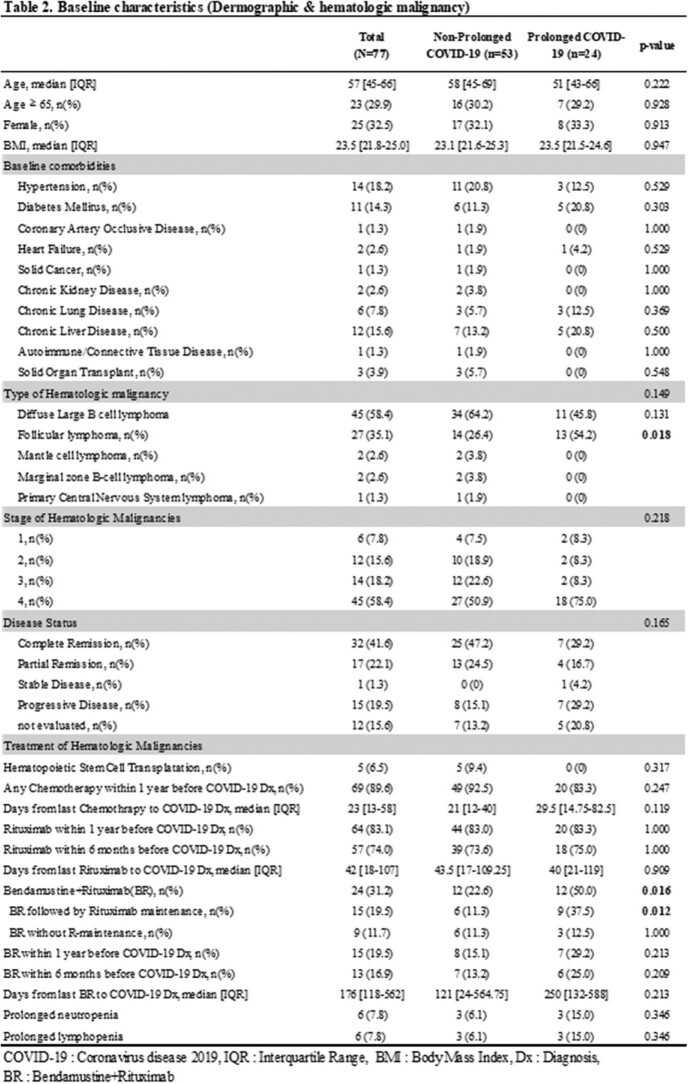

**Figure:**
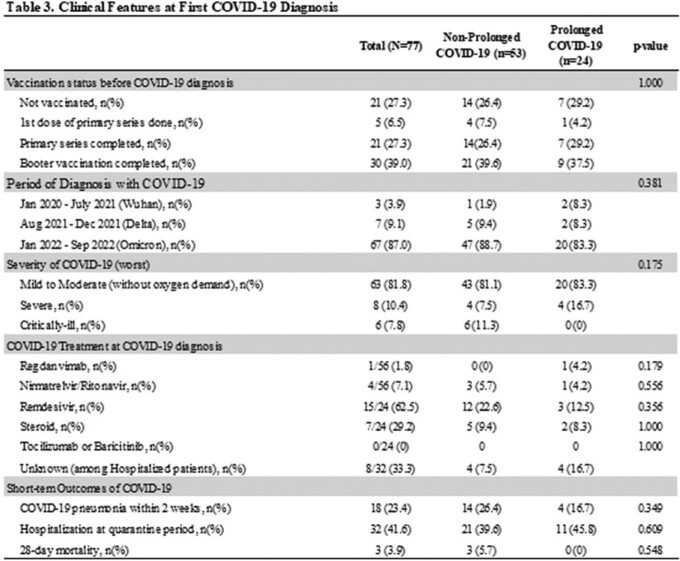

**Conclusion:**

In this study, patients with lymphoma who have received certain treatments may have a higher risk of developing COVID-19 pneumonia due to prolonged infection. Further study is needed to understand the immune responses in this patient group and find treatment methods.

**Figure:**
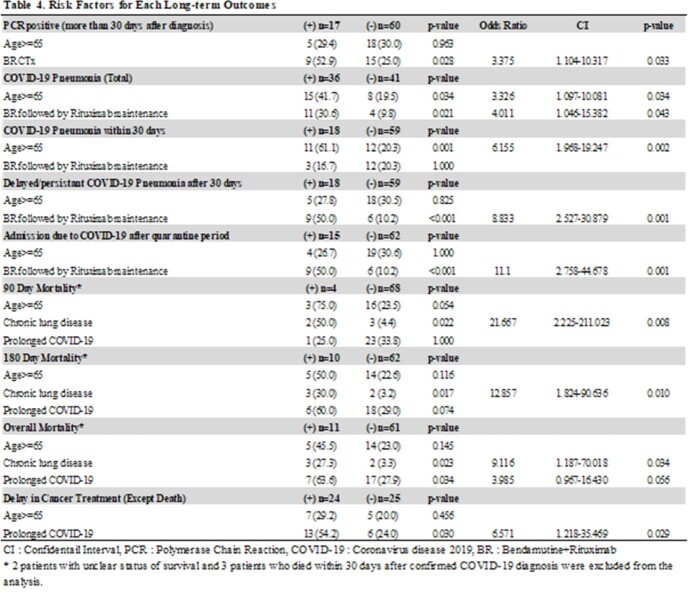

**Disclosures:**

**All Authors**: No reported disclosures

